# Prevalence and correlates of female sexual dysfunction and sexual distress in reproductive-aged women: a systematic review and meta-analysis

**DOI:** 10.1186/s12905-025-03960-4

**Published:** 2025-09-29

**Authors:** Fatemeh Heshmatnia, Marzieh Azizi, Hamed Milani, Roya Nikbakht, Mohsen Kheiri, Hadis Tolomehr, Zohreh Shahhosseini

**Affiliations:** 1 Department of Nursing and Midwifery, Islamic Azad University, Se.C, Semnan, Iran; 2 Department of Midwifery, Sexual and Reproductive Health Research Center, Mazandaran University of Medical Sciences, Sari, Iran; 3 School of Medicine, Mazandaran University of Medical Sciences, Sari, Iran; 4 Department of Biostatics and Epidemiology, Faculty of Health, Mazandaran University of Medical Sciences, Sari, Iran; 5 Student Research Committee, School of Pharmacy, Mazandaran University of Medical Sciences, Sari, Iran; 6 Student Research Committee, School of Paramedical Sciences, Mazandaran University of Medical Sciences, Sari, Iran

**Keywords:** Female sexual dysfunction, Sexual distress, Prevalence, Risk factors, Reproductive-aged

## Abstract

**Background:**

Female sexual dysfunction (FSD) is defined as any dissatisfaction with sexual function domains such as sexual desire/arousal disorder, orgasmic disorders, and genital-pelvic pain/penetration disorder that in some cases leads to sexual distress (SD).

**Objective:**

This systematic review and meta-analysis aimed to examine the prevalence and associated factors of FSD and SD among healthy reproductive-aged women.

**Methods:**

In this systematic review and meta-analysis, Google Scholar and electronic databases such as Scopus, ScienceDirect, PubMed, Web of Science, and the Iranian database including the Scientific Information Database (SID), were searched and the publication year of the included articles were limited to January 1, 2015, through January 1, 2024 to identify studies that assessed FSD and SD among reproductive-aged women. The quality of the included studies was evaluated using the Newcastle-Ottawa Scale for cross-sectional and cohort studies.

**Results:**

Twenty studies were included in this review. According to the results of 18 studies, the prevalence of FSD ranged between 20.6% and 95.0%. In addition, the prevalence of sexual desire disorder (SDD) was estimated between 8.0% and 91.0% based on the results of 16 studies. The prevalence of arousal disorder (9.0–91.0%), orgasm disorder (7.9–93.0%), lubrication problems (9.3–99.0%), pain disorder (8.2–99.0%), and satisfaction (21.4–86.0%). SD prevalence was 31.8–83.4%. Meta-analysis revealed a pooled FSD prevalence of 47.81% (95% CI: 39.19–56.43%). The factors related to FSD were classified into five main categories: sociodemographic, reproduction, interpersonal, psychological, and medical factors. Also, the associated factors of SD were classified into four categories: sociodemographic, interpersonal, psychological, and medical factors. The risk of bias for all of the included studies was good.

**Conclusion:**

The results of this study showed that reproductive-aged women are at high risk of FSD and SD due to various factors. As the literature regarding SD among the reproductive-aged population was limited, conducting high-quality cross-sectional studies with representative samples and using validated questionnaires are required to provide more precise data regarding its prevalence and associated factors across diverse cultural contexts.

**Trial registration:**

PROSPERO; Registration no. CRD42024491942.

**Supplementary Information:**

The online version contains supplementary material available at 10.1186/s12905-025-03960-4

## Background

Sexuality is a natural and complex element of human behavior, influenced by various physiological, psychological and social factors [1–3]. Women at different stages of life, including in reproductive-aged, may be exposed to problems in their sexual function, which negatively affects their sexual health and emotional well-being [4]. According to the World Health Organization, sexual health refers not only to the absence of disease, dysfunction and disability but also to the physical, emotional, mental, and social aspects of sexuality [5]. Based on the Diagnostic and Statistical Manual of Mental Disorders, Fifth Edition, Text Revision (DSM-5-TR), female sexual dysfunction (FSD) is defined as any dissatisfaction with sexual function domains such as sexual desire/arousal disorder, orgasmic disorders, and genital-pelvic pain/penetration disorder that leads to considerable sexual distress (SD) [6, 7]. In the global context, FSD prevalence varies by community, ranging from 35.4 to 62.1% [8, 9]. Based on a systematic review and meta-analysis that examined the prevalence of FSD in reproductive-aged women, the prevalence of pain, sexual arousal disorder, sexual desire, vaginal lubrication and orgasm disorder were 39.08, 48.21, 50.70, 37.60, and 40.16% calculated respectively [10].

Studies showed that several factors, such as age, anatomical and neurological factors, hormonal issues, urinary dysfunction, drug intake, psychological difficulties, and sociocultural factors, are associated with FSD among reproductive-aged women [11, 12]. Furthermore, a survey in Germany indicated sexual assault, poor physical health, abortion, and vaginal delivery were found among significant associated factors of FSD [13]. Other factors, such as low family income and dissatisfaction with a spouse’s sexual ability, were considered significant independent factors in a Sample of Saudi Women [14]. FSD negatively affects women’s quality of life and physical well-being [15, 16], lowers self-esteem, and strains family relationships, resulting in frustration, anxiety, and depression [17].

SD, a critical diagnostic criterion for FSD that refers to a persistent or recurrent state of emotional suffering directly linked to difficulties in sexual function, response, or satisfaction. It encompasses negative affective and cognitive reactions such as embarrassment, guilt, shame, frustration, anxiety, fear, anger, or diminished self-worth that arise from perceived or actual impairments in sexual desire, arousal, orgasm, or pain during sexual activity. The presence of significant SD is a crucial criterion in the diagnostic process of sexual dysfunction [18]. Epidemiological studies in Europe have shown that 46–65% of women with sexual problems suffer from SD [19, 20]. Many factors, such as age, employment, socioeconomic, and general health status, affect women’s SD. In general, SD affects all aspects of people’s lives and can lead to marital disruption and decreased quality of life [21].

The literature review showed that although some systematic reviews and meta-analyses were conducted regarding the predictors of FSD among reproductive-aged women, these studies assessed studies published before 2015 [10, 22]. While these earlier reviews provide foundational knowledge, more recent primary studies have emerged with updated data on FSD prevalence and correlates in reproductive-aged populations. For instance, a cross-sectional study by Zeleke et al. among 424 reproductive-aged women in Ethiopia reported a 32.1% prevalence of FSD, highlighting the continued relevance of this issue in contemporary populations [23]. In addition, SD was not assessed among reproductive-aged women. Despite the high prevalence of FSD with or without SD, it is often neglected, under-identified, and untreated by healthcare professionals [24, 25]. FSD and SD prevalence rates and associated factors should be analyzed epidemiologically to guide clinical interventions and inform health policy decisions addressing women’s sexual health. Thus, this systematic review and meta-analysis aimed to examine the prevalence and associated factors of FSD and sexual distress SD among healthy reproductive-aged women.

## Methods

### Protocol and registration

This systematic review and meta-analysis was reported according to the Preferred Reporting Items for Systematic Reviews and Meta-Analyses (PRISMA) statements 2020 [26]. Before conducting this study, its protocol was registered in the International Prospective Register of Systematic Reviews (PROSPERO; Registration no. CRD42024491942, no amendments were made post-registration) and is available from: https://www.crd.york.ac.uk/PROSPERO/view/CRD42024491942.

### Search strategy

To collect the data for this systematic review, we searched Google Scholar and electronic databases such as Scopus, ScienceDirect, PubMed and Web of Science. In addition, to collect the data for this systematic review, we searched the Iranian database, including the Scientific Information Database (SID; a national database indexing Persian-language peer-reviewed journals in medical and social sciences) to identify studies that assessed FSD and SD among reproductive-aged women. In addition, to minimize language bias by incorporating locally relevant studies, which aligns with PRISMA’s equity considerations for systematic reviews. The latest search process by authors was performed in a time bound of October 31, 2023 to April 29, 2024.

We limited the publication year of the included articles to January 1, 2015, through January 1, 2024. The search was performed by two authors (F.H. and M.A.) independently. In others words, our review specifically focuses on studies published from 2015 to 2024 to capture the most current epidemiological data. This timeframe allows us to analyze newer evidence that may reflect evolving sociocultural attitudes, updated diagnostic criteria (DSM-5-TR), and modern healthcare contexts affecting sexual health.

To search in mentioned databases, first, Medical Subject headings were used to extract search terms including (“Sexual dysfunction” [Mesh]) OR (female sexual dysfunction [Mesh]) OR (psychosexual disorders [Mesh]) OR (female sexual disorders [Mesh]) OR (hypoactive sexual desire disorders [Mesh]) OR (female low desire disorder [Mesh]) OR (sexual arousal disorder [Mesh]) OR (orgasmic disorders [Mesh]) OR (dyspareunia [Mesh]) OR (genito-pelvic pain/penetration disorder [Mesh]) OR (sexual distress [Mesh]) OR (female sexual distress [Mesh]) AND (reproductive-aged women [Mesh]) AND [(prevalence [Mesh]) OR (rate[Mesh]) OR (incidence[Mesh]). We searched other databases according to the specific search guidelines for advanced searches provided by each database and the search strategy is listed in Table 1. In addition, all reference lists of included studies were manually searched to ensure that all additional published articles were identified. The reference manager software EndNote 21 was used to collect references and eliminate duplicate records.

**Table 1:** The search strategy in databases

Last search date	The latest search was performed from “15 October 2023 to 29 April 2024.”		
Database	Search strategy	Number of extracted studies	Search Filters
PubMed	(sexual dysfunction[TIAB]) OR (sexual disorder[TIAB]) OR (female sexual dysfunction [TIAB]) OR (hypoactive sexual desire disorder[TIAB]) OR (low sexual desire[TIAB]) OR (sexual arousal disorder[TIAB]) AND (orgasm disorder[TIAB]) OR (dyspareunia [TIAB]) OR (genito-pelvic pain/penetration disorder [TIAB]) OR (female sexual distress[TIAB]) AND (reproductive-aged [TIAB]) OR (reproductive [TIAB]) AND (prevalence [Mesh]) OR (rate[Mesh]) OR (incidence[Mesh]).	4033	-Text availability: Full-text -Article type: Observational studies
Scopus	TITLE-ABS-KEY (sexual dysfunction OR sexual disorder OR hypoactive sexual desire disorder OR sexual desire disorder OR sexual distress OR female sexual distress) AND (reproductive aged OR reproductive-aged women)	1287	-Subject areas: Medicine -Document types: Article -Source type: Journal
WOS	TS=(sexual dysfunction OR sexual disorders OR hypoactive sexual desire disorder OR sexual desire disorder OR sexual distress OR female sexual distress) AND (reproductive aged OR reproductive-aged women)	4056	-Document types: Article -Publication years = 2015–2024 - Open access = All open access
ScienceDirect	Female sexual dysfunction OR sexual distress AND reproductive-aged women	2701	Years: 2015–2024 -Article type: Research article -Subject area: Medicine - Language: English Access type: Open Access & Open archive
Scientific Information Database (SID)	Sexual dysfunction OR sexual distress AND reproductive-aged women	716	Topic: Sexual dysfunction
Google Scholar	(“sexual dysfunction” OR “sexual disorder” OR “sexual desire disorder” OR “sexual distress” OR “female sexual distress”) AND (“reproductive-aged” OR “reproductive-aged women”)	1340	-Year of publication: 2015–2024 -Sort by relevance -Keywords in the title of the article

### Data selection

In this systematic review, inclusion criteria were as follows:

- 1- Cross-sectional and cohort studies reported the prevalence of FSD in at least one domain of FSD according to DSM-IV or DSM-5-TR, or SD separately or FSD associated with SD among reproductive-aged women (15–49 years). Notably, we included only cross-sectional and cohort studies for the following reasons:

Cross-sectional studies provide a “snapshot” of FSD/SD prevalence and correlates at a specific time, which is essential for estimating the burden of these conditions in reproductive-aged women. Cohort studies (though rare in this field) allow for tracking changes in sexual function over time, offering insights into potential causal relationships.

- 2- Studies published in scientific journals between January 1, 2015, through January 1, 2024.
- 3- Observational studies with available information regarding related factors of FSD and SD among reproductive-aged women.

In contrast, study designs, including case reports, case series, letters, narratives, scoping and systematic reviews, meta-analysis and randomized controlled trials (RCTs) regarding FSD and/or SD among healthy menopause women or healthy reproductive-aged women, studies which conducted regarding the FSD or SD on women with chronic physical and psychiatric disorders and also on menopause women were excluded from this study.

In addition, while this systematic review adheres to PRISMA 2020 guidelines for reporting, we excluded gray literature (e.g., conference abstracts, dissertations, unpublished reports) for the following reasons: Gray literature frequently lacks rigorous methodological details such as standardized sampling approaches, validated measurement tools, and comprehensive statistical analyses, making proper quality assessment challenging. The lack of consistent diagnostic criteria for FSD and SD, particularly those defined in the DSM-5-TR and measured using validated tools such as the FSFI and FSDS-R, further exacerbates these limitations when relying on non-peer-reviewed sources.

### Type of outcome measure

FSD was defined per DSM-5-TR criteria as clinically significant difficulty in one or more of the following domains for a minimum duration of approximately 6 months: (1) absent/reduced interest in sexual activity, (2) reduced arousal (subjective or genital), (3) difficulty achieving orgasm, or (4) pain during intercourse, accompanied by significant distress. SD was assessed using validated tools (e.g., FSDS-R) measuring distress specific to sexual function.

The primary outcome of this study was to systematically assess the prevalence of at least one domain of FSD (based on DSM-IV or DSM-5-TR criteria), either as an independent condition or in association with SD, among healthy reproductive-aged women. The secondary outcome was to evaluate factors associated with FSD, SD, or FSD comorbid with SD in this population.

### Data extraction

The titles and abstracts of all included studies were evaluated for their relevance. At this stage, irrelevant abstracts were retained until the full text of the article was reviewed. After carefully reading the full text of the selected articles, the required information was extracted into descriptive tables and cross-checked by Z.Sh. Two investigators (F.H. and M.A.) independently assessed each publication for eligibility and compared the results. The final decision is based on discussions with a third author (Z.Sh.) if there is a discrepancy in their assessment. A piloted data extraction form was used, capturing key details, such as the first author, the country, the year of study, the study design, the mean ± SD of age or age groups, the sampling method, the sample size, the instruments, and the prevalence of FSD/SD and associated factors.

### Methodologic quality assessment

One of the most widely used scales for evaluating the quality and risk of bias in observational studies is the Newcastle-Ottawa Scale (NOS) [27]. The NOS for cohort studies evaluates three quality parameters (selection, comparability, and outcome) divided across nine specific items; it slightly differs when scoring cross-sectional, case-control, and cohort studies. The scoring for cohort studies is as follows: good quality (3 or 4 stars in the selection domain, 1 or 2 stars in the comparability domain, and 2 or 3 stars in the outcome/exposure domain), fair quality (2 stars in selection domain, 1 or 2 stars in comparability domain, and 2 or 3 stars in outcome/exposure domain), and poor quality (0 or 1 star in selection domain, 0 stars in comparability domain, or 0 or 1 star in outcome/exposure domain [28, 29]. In cross-sectional studies, the NOS evaluates three quality parameters (selection, comparability, and outcome) divided across eight specific items; each item on the scale is scored from one point, except for the comparability parameter, which scores up to two points. Thus, the maximum for each study is 9, and studies with scores < 5 points are identified as having a high risk of bias [28, 29]. Two reviewers independently assessed bias using the NOS. Disagreements were resolved through discussion. Results informed sensitivity analyses (e.g., excluding lower-quality studies).

## Results

### The search results and selection strategy

**Fig. 1:**
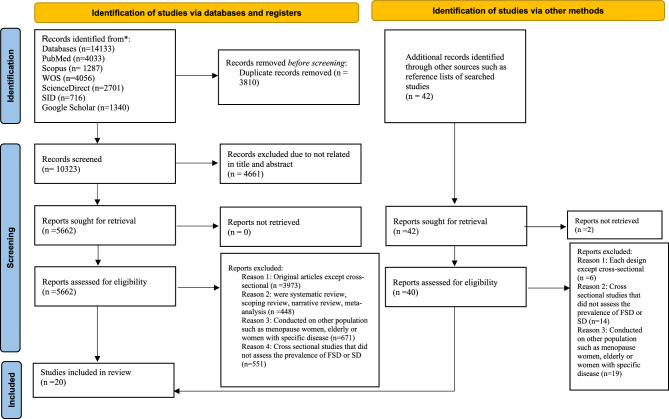
The PRISMA diagram for the search of records and study selection

The search results in databases and screening processes are shown in Fig. 1. The search resulted in 14,133 articles (PubMed (*n* = 4033), Scopus (*n* = 1287), WOS (*n* = 4056), ScienceDirect (*n* = 2701).

SID (*n* = 716) and Google Scholar (*n* = 1340)).

After removing duplicate articles (*n* = 3,810), 10,323 articles remained. Of these, 4,661 were excluded based on title and abstract screening. During full-text assessment we excluded: 3,973 case-control and randomized controlled trials, 448 review articles (scoping, narrative, systematic reviews and meta-analyses), 671 studies focusing on other populations (menopausal women, elderly, or reproductive-aged women with specific diseases), 551 cross-sectional studies that did not assess the prevalence of FSD or SD. Additionally, we examined reference lists of searched studies and identified 42 potentially relevant articles. Two of these could not be retrieved, and 39 of the remaining 40 were excluded for various reasons. Ultimately, 20 studies met our inclusion criteria and were included in this systematic review (Fig. 1).

### The characteristics of the included studies

The results of the included studies are presented in Table 2. Of the 20 studies, seven were conducted in Iran [8, 30–35], two in China [36, 37], two in Egypt [38, 39], one in Turkey [40], one in Brazil [41], one in Australia [19], one in Ethiopia [23], one in Singapore [42], one in Kenya [43], one study in Nigeria [44], one study in Cameroon [24], and one in Hong Kong [15]. The included studies were published from 2015 to 2023. The studies had various sample sizes (187 and 6,986) and considered 36,777 reproductive-age women. Of the 20 included studies, 19 had cross-sectional studies, and one had a prospective cohort design [42]. Random sampling method was used in four studies [15, 23, 34, 39], convenience sampling method was applied in nine studies [8, 24, 33, 35, 36, 38, 41, 42, 44], two studies used two-stage cluster sampling [30, 31], one study used single-stage cluster sampling [40], two studies used multi-stage cluster sampling [32, 37], consecutive sampling method was used in one study [43] and one study census was used for data collection [19]. In 16 studies, the Female Sexual Function Index (FSFI) was used to assess the prevalence of FSD [8, 15, 23]^32^– [37]^39^– [45]. In five studies, the Female Sexual Distress Scale-Revised (FSDS-R) was used to evaluate the prevalence of SD [19, 30, 31, 41, 46], and Profile of Female Sexual Function (PFSF) was used in one study [19].

**Table 2:** Characteristics of included studies in the systematic review (*n* = 20)

First author, country	Year of study	Study design	Mean ± SD or age groups (Year)	Sampling method	Sampling size	Instruments
Hamzehgardeshi, Iran [31]	2023	Cross-sectional	16–49	Two-stage cluster	1000	FSDS-R
Zeleke, Ethiopia [23]	2023	Cross-sectional	< 30 - >40a	Systematic random	424	FSFI
Halle-Ekane Ge, Cameroon [24]	2021	Cross-sectional	> 16	Convenience	405	FSFI
Loy, Singapore [42]	2021	Prospective cohort	18–45	Convenience	513	FSFI
Ju, China [36]	2021	Cross-sectional	32–63	Convenience	524	FSFI
Hamzhgardeshi, Iran [30]	2020	Cross-sectional	16–49	Two-stage cluster	1000	SIDI-F, FSDS-R
Zheng, J, Australia [19]	2020	Cross-sectional	18–39	Census	6986	PFSF, FSDS-R
Rezaie-Chamani, S, Iran, 2020 [32]	2020	Cross-sectional	15–49	Multi-stage cluster sampling	400	FSFI
Cerentini, Brazil [41]	2020	Cross-sectional	22.44 ± 3.88	Convenience	187	FSFI, FSDS-R
Yilmaz, Turkey [40]	2020	Cross-sectional	15–49	Single-stage cluster	1323	FSFI
Butt, Kenya [43]	2019	Cross-sectional	32.3	Consecutive	566	FSFI
El-Kashif, Egypt [39]	2019	Cross-sectional	15–51	Random	780	FSFI
Ahmed, Egypt [38]	2017	Cross-sectional	20–45	Convenience	241	FSDS-R
Lou, China [37]	2017	Cross-sectional	> 20	Multiple‑stage cluster sampling	4697	FSFI
Alidost, Iran [33]	2017	Cross-sectional	16–43	Convenience	300	FSFI
Shittu, R.O., Nigeria [44]	2017	Cross-sectional	15–49	Convenience	300	FSFI
Jafarzadeh, Iran [8]	2016	Cross-sectional	15->39	Convenience	264	FSFI
Javadifar, Iran [34]	2016	Cross-sectional	15–45	Random	800	FSFI
Zhang, Hong Kong [45]	2015	Cross-sectional	19–50	Random stratified	1,518	FSFI
Sadat, Iran [35]	2015	Cross-sectional	18–49	Convenience	200	FSFI

*FSFI* Female Sexual Function Index, FSDS-R Female Sexual Distress Scale-Revised, PFSF Profile of Female Sexual Function

### The prevalence of FSD and its domains in included studies

The prevalence of FSD and its domains among reproductive-aged women is shown in Table 3. FSD domains were categorized according to the included studies reporting such as low sexual desire, arousal disorder, orgasm disorder, lubrication problems, pain disorder, satisfaction, and SD. According to the results of 18 studies, the prevalence of FSD ranged between 20.6% and 95.0%. In addition, the prevalence of sexual desire disorder (SDD) was estimated between 8.0% and 91.0% based on the results of 16 studies. The prevalence of arousal disorder (9.0–91.0%), orgasm disorder (7.9–93.0%), lubrication problems (9.3–99.0%), pain disorder (8.2–99.0%), satisfaction (21.4–86.0%) were estimated. The prevalence of SD was assessed in four studies and ranged between 31.8% and 83.4% in reproductive-aged women [19, 31, 38, 41]. According to the DSM-5, hypoactive sexual desire disorder (HSDD) is replaced instead of low sexual desire and arousal disorder, and genito-pelvic pain disorders are replaced instead of pain disorder. Still, in all included studies, HSDD was not reported.

**Table 3:** Prevalence of FSD, its domains, SD and related factors in reproductive-aged women according to the included studies

Prevalence (%)											
Author, country, year (Ref.)	FSD	SDD		Arousal disorder	Orgasm disorder	Lubrication problems	Pain disorder	Satisfaction	SD	FSD associated with SD	Related factors of FSD or SD
Hamzehgardeshi, Z, Iran, 2023 [31]	-	24.8		-	39.9	49.4	8.2	21.4	31.8	-	Satisfaction with marriage (OR = 0.46, *p* = 0.001), history of infertility (OR = 2.73, *p* < 0.01), fear of STDs (OR = 2.70, *p* < 0.01), pain during sexual intercourse (OR = 2.17, *p* = 0.01), premature ejaculation disorders in the partner (OR = 2.00, *p* < 0.05) associated with SD.
Zeleke, F, Ethiopia, 2023 [23]	32.1	58.5		91.0	47.8	89.3	39.3	42.8		-	BMI (OR = 3.6, *p* < 0.01), history of pelvic surgery (OR = 3.5, *p* < 0.01), marriage satisfaction (OR = 3.9, *p* < 0.05), satisfaction of spouses’ sex ability (OR = 3.1, *p* < 0.01), breastfeeding (OR = 3.3, *p* < 0.05), and vaginal delivery (OR = 3.7, *p* < 0.01) associated with FSD.
Halle-Ekane Ge, Cameroon, 2021[24]	42.0	29.1		21.2	42.0	-	46.9	-		-	Primary educational level (*p* < 0.005), history of sexual assault (*p* = 0.012), and poor physical health (*p* = 0.012) associated with FSD.
Loy, S, Singapore, 2021[42]	58.9	74.0		41.0	36	91.5	41.0	23.5		-	Higher BMI (OR = 1.08, *p* < 0.01) and anxiety (OR = 2.72, *P* < 0.01) were associated with FSD.
Ju, R, China, 2021 [36]	40.9	43.7		32.2	28.3	17.2	14.9	25.0		-	Higher age (OR = 1.071, *p* < 0.001), rural residential environment (OR = 2.21, *p* < 0.001), dissatisfaction of marital relations (OR = 2.32, *p* = 0.002), secondary educational status (OR = 2.07, *p* = 0.026), smoking (OR = 4.12, *p* = 028) and chronic disease (OR = 1.59, *p* = 0.032)
Hamzehgardeshi, Z, Iran, 2020 [30]	-	-		-	-	-	-	-		-	Lower age at first intercourse (*p* < 0.001), duration of marriage (*p* < 0.001), and level of satisfaction with income (*p* < 0.001) were significantly associated with both LSD and HSDD, and BMI (*p* < 0.01) were just predictors of LSD (*p* < 0.001).
Zheng, J, Australia, 2020 [19]	20.6	8.0		9.0	7.9				50.2	20.6	Psychotropic medication (OR = 1.76, *p* < 0.001) was significantly associated with FSDs. Psychotropic medication (OR = 1.94, *p* < 0.001), sexual inactivity (OR = 1.90, *P* < 0.001), paid employment (OR = 1.20, *p* < 0.01), being a smoker (OR = 1.24, *p* < 0.02), alcohol consuming (OR = 1.16, *p* < 0.03) and infertility treatment (OR = 2.31, *p* < 0.001) was significantly associated with SD among participants.
Cerentinie, TM, Brazil, 2020 [41]	23.0	88.4		90.7	93.0	93.0	97.7	58.1	83.4	17.1	In this study the logistic regression did not highlight the existence of FSD predictive factors in the samples.
Rezaie-Chamani, Iran, 2020 [32]	34.3	-		-	-	-	-	-		-	Higher age (*p* = 0.001), secondary educational level (*p* = 0.002), no more than one-time sex (*p* = 0.01), and lower knowledge of sexual infections (*p* = 0.02) associated with FSD.
Yilmaz, Turkey, 2020 [40]	40.5	-		-	-	-	-	-		-	Poor marital adjustment (OR = 4.06, *p* < 0.001) and poor mental health (OR = 2.74, *p* < 0.001), lack of social insurance (OR = 1.85, *p* = 0.020), chronic disease of the spouse (OR = 1.67, *p* = 0.016), perception of deficient knowledge on sexuality-related topics (OR = 2.00, *p* = 0.002), perceived sexual problems (OR = 2.95, *p* = 0.001), and increasing duration of marriage (OR = 3.53, *P* < 0.001) were also associated with FSD.
Butt, M, Kenya, 2019 [43]	38.7	-		-	-	-	-	-		-	Hormonal contraception (OR = 2.69, *p* < 0.0001) was the only significant associated factor of FSD.
El-Kashif, M, Egypt, 2019 [39]	53.1	67.3		55.8	51.2	51.5	51.5	43.0		-	Low education (OR = 4.8, *p* < 0.05), genital tract inflammation (OR = 3.4, *p* < 0.05), and stress (OR = 2.3, *p* < 0.05) were associated with FSD.
Ahmed, MR, Egypt, 2017 [38]	58.1	37.9		30.7	45.0	32.9	34.3	47.9	36.4	-	Marriage duration (*p* = 0.001) was associated with FSD.
Lou, WJ, China, 2017 [37]	63.3	46.5		80.1	29.9	32.4	31.6	33.0		-	Age (OR = 1.05), dissatisfaction with the spouse’s sexual ability (OR = 3.52), poor marital adjustment (OR = 2.08), spouse sexual difficulties (OR = 1.72), living in a rural area (OR = 1.29), lower education (OR = 3.44), chronic disease (OR = 1.53), vaginal delivery (OR = 2.28), dissatisfaction with married life (OR = 1.47), previous pelvic surgery (OR = 1.60)
Alidost, F, Iran, 2017 [33]	65.0	46.3		53.0	48.3	40.7	48.3	22.7		-	Quality of life (*p* < 0.01), age (*p* = 0.001), and prenatal anxiety (*p* = 0.04) affected the FSD.
Shittu, R.O., 2017, Nigeria [44]	95.0	91.0		89.0	92.0	99.0	99.0	86.0		-	The associated factors of FSD” were not investigated in this study.
Jafarzadeh, R, 2016, Iran [8]	62.1	49.2		43.2	38.6	36.0	35.2	26.1		-	Employment status of spouse (*p* = 0.03), use of medications (*p* = 0.01) and education level of women (*p* = 0.01) had significant effects on FSFI scores.
Javadifar, N, 2016, Iran [34]	47.75	21.9		22.6	16.5	-	38.9	22.0		-	No delivery (OR = 4.12, *p* = 0.002), NVD (OR = 2.13, OR = 0.008), higher BMI (OR = 1.85, *p* = 0.006) and rural residential location associated with FSD.
Zhang, H, 2015, Hong Kong [45]	25.6	10.6		10.5	8.8	9.3	8.4	-		-	Neutral attitude to sex (OR = 2.02, *p* < 0.05). have an abortion (OR = 1.62, *p* < 0.01), have no more than once time sex monthly (OR = 1.95, *p* < 0.01), neutral (OR = 2.09, *p* < 0.01) and traditional attitude to sex (OR = 1.66, *p* < 0.01), average or poor health (OR = 1.67, *p* < 0.05), associated with FSD.
Sadat, Z, Iran, 2015 [35]	60.0	39.0		37.0	24.5	28.8	19.5	22.5		-	Older age (*p* = 0.006), longer duration of the marriage (*p* = 0.005), primary educational level (*p* = 0.027), depression (*p* < 0.001), anxiety (*p* = 0.002) and stress (*p* < 0.001) associated with FSD.

*SDD* Sexual desire disorder, *FSD* Female sexual dysfunction, *SD* Sexual distress, *STDs* Sexually transmitted diseases, *BMI* Body mass index, *NVD* Normal vaginal delivery, *OR* Odds ratio

### The associated factors of FSD among reproductive-aged women

The factors related to FSD among reproductive-aged women are listed in Table 3. These associated factors were classified into five main categories: sociodemographic, reproduction, interpersonal, psychological, and medical factors.

In 15 studies the sociodemographic associated factors of FSD were reported. The sociodemographic factors such as higher BMI [23, 34, 42] (OR = 1.85, *p* = 0.006 [32]; OR = 3.6, *p* < 0.01 [40]; OR = 1.08, *p* < 0.01 [41]), higher age [32, 33]^35^– [37] [OR = 1.071, *p* < 0.001 [30]; OR = 1.05, *p* = 0.001 [31]; OR = 1.05, *p* = 0.006 [33–35]), longer marriage duration [30, 35, 39, 40], rural residential environment [34, 36, 37] (OR = 2.21, *p* < 0.001 [32]; OR = 1.29, *p* = 0.034 [35]), primary or secondary educational status [8, 24, 32]^35^– [37, 43] (primary: *p* < 0.005 [7, 22]; secondary: OR = 2.07, *p* = 0.026 [30]^33^– [35, 42]), smoking (OR = 4.12, *p* = 0.028 [36]), no specified spouse’s employment status [8], level of income satisfaction [30], lack of social insurance (OR = 1.85, *p* = 0.020 [40]), poor knowledge of sexual topics such as sexual intercourse and sexual infections [32, 40], neutral and traditional attitude to sex (OR = 2.09, *p* < 0.01 [15]) were significantly associated with FSD among reproductive-aged women according to the included studies.

The reproductive associated factors of FSD were investigated in five studies. The reproduction factors associated with FSD were history of abortion (OR = 1.62, *p* < 0.01 [15]), Nulliparity (OR = 4.12, *p* = 0.002 [34]), vaginal delivery [34, 37] (OR = 3.7, *p* < 0.01 [32]; OR = 2.28, *p* = 0.035 [35]), (OR = 2.69, *p* < 0.0001 [43]), and breastfeeding (OR = 3.3, *p* < 0.05 [23]).

In eight included studies the interpersonal associated factors of FSD were assessed. Factors such as satisfaction with marriage (OR = 0.46, *p* = 0.001 [23]), dissatisfaction of spouse’s sex ability [23, 37], dissatisfaction of marital relations [36, 37], poor marital adjustment (OR = 4.06, *p* < 0.001 [37, 40]), sexual inactivity or no more than one-time sexual relationship monthly (OR = 1.95, *p* < 0.01 [15, 32]), genital tract inflammation (OR = 3.4, *p* < 0.05 [39]), dyspareunia, and partner’s premature ejaculation (OR = 2.00, *p* < 0.05 [31]) were the significant interpersonal factors associated with FSD among participants.

The psychological risk factors of FSD were evaluated in five included studies. Psychological factors such as fear of STDs, poor mental health (OR = 2.74, *p* < 0.001 [40]), history or current anxiety (OR = 2.72, *p* < 0.01 [33, 35, 42]), stress (OR = 2.3, *p* < 0.05 [35, 39]), depressive disorders [35], and history of sexual assault [24] were associated significantly with FSD among reproductive-aged women.

According to the results of four included studies, the significant medical-associated factors of FSD among participants were women or their spouse’s chronic diseases (OR = 1.59, *p* = 0.032 [36, 37]), history of pelvic surgery (OR = 3.5, *p* < 0.01 [23, 35, 37, 40]), genital tract inflammation, and use of psychotropic medications (OR = 1.76, *p* < 0.001 [19]).

### The associated factors of SD among reproductive-aged women

The related factors of SD among reproductive-aged women are listed in Table 3. Two studies of 17 included studies only assessed SD-associated factors among participants [19, 31]. These associated factors were classified into four main categories: sociodemographic, interpersonal, psychological, and medical factors.

The sociodemographic factors included paid employment (OR = 1.20, *p* < 0.01) [19], being a smoker (OR = 1.24, *p* < 0.02) [19] and alcohol consuming (OR = 1.16, *p* < 0.03) [19] which were significantly associated with SD according to one included studies.

According to the results of two included studies, the interpersonal factors included satisfaction with marriage [31], spouse’s premature ejaculation disorder [31], pain during sexual intercourse [31] and sexual inactivity [19], which were significantly associated with SD among participants.

Fear of STDs was the only psychological factor associated with SD [31]. Medical factors including a history of infertility [31] or infertility treatments [19] and psychotropic medications [19] were associated with SD according to the two included studies.

#### Meta-analysis results

**Fig. 2:**
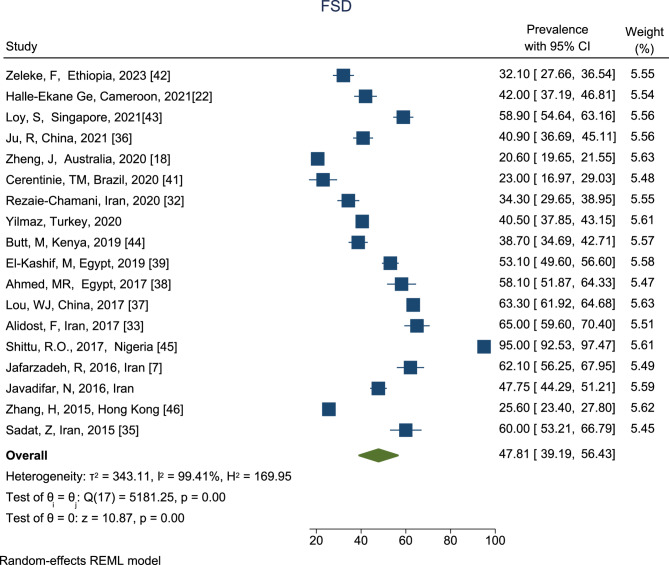
The prevalence of FSD among reproductive-aged women

The prevalence of FSD among reproductive-aged women is shown in Fig. 2. According to the results, the highest prevalence of FSD was in the Shittu et al. study [44] (95.00%, 95% CI: 92.53–97.47%); the lowest was in Zheng et al. (20.60%, 95% CI: 19.65–21.55%) study in Australia [19]. Based on the combined results of 18 studies, the total prevalence of FSD among reproductive-aged with a CI of 95% and based on the REM was estimated to be 47.81% (95% CI, 39.19–56.43%).

**Fig. 3:**
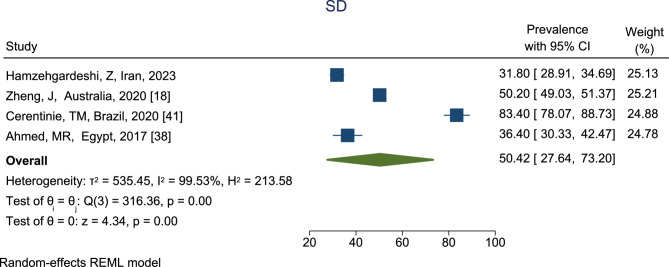
The prevalence of SD among reproductive-aged women

The prevalence of SD among reproductive-aged women is shown in Fig. 3. According to the results, the highest prevalence of SD was in the Cerentinie et, al study [41] in Brazil, which was reported as 83.40% (95% CI, 78.07 − 88.73%), and the lowest prevalence was in the Hamzehgardeshi et al. study [31] in Iran which was reported as 31.80% (95% CI, 28.91–34.69%). Based on the combined results of four studies, the total prevalence of SD among reproductive-aged with a CI of 95% and based on the REM was estimated to be 50.42% (95% CI, 27.64–73.20%).

**Fig. 4:**
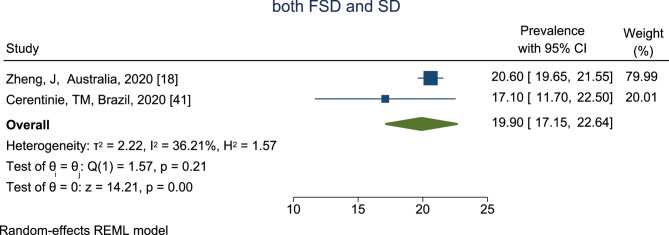
The prevalence of FSD and associated SD among reproductive-aged women

The prevalence of FSD associated with SD among reproductive-aged women is shown in Fig. 4. According to the results, the highest prevalence of FSD associated with SD was in the Zheng et, al study [19] in Australia, which was reported as 20.60% (95% CI, 19.65 − 21.55%), and the lowest prevalence was in Cerentinie et, al study [41] in Brazil which was reported as 17.10% (95% CI, 11.70–22.50%). Based on the combined results of two studies, the total prevalence of FSD associated with SD among reproductive-aged with a CI of 95% and based on the REM was estimated to be 19.90% (95% CI, 17.15–22.64%).

### Quality assessment

The quality of the 19 cross-sectional included studies was evaluated using the NOS for cross-sectional studies. All of these studies had good quality (> 5 points). The quality of only one cohort study was also assessed using NOS for cohort study and had good quality. The details of the studies’ scoring are presented in Tables 4 and 5.

**Table 4:** The quality assessment of the included studies using the Newcastle Ottawa scale for cross-sectional studies

	Selection					Compatibility	Outcome		
Row	First author, year (Ref)	Representativeness of samples	Sample size	Non-respondent	Ascertainment of the exposure	The subjects in different outcome groups are comparable based on the study design or analysis. Confounding factors are controlled	Assessment of the outcomes	Statistical test	Score (0–10 stars)
1	Hamzehgardeshi, 2023 [31]	b (*)	a (*)	c	a (**)	a (*)	c (*)	a (*)	7
2	Zeleke, 2023 [23]	a (*)	a (*)	a (*)	a (**)	a (*)	c (*)	a (*)	8
3	Halle-Ekane Ge, 2021 [24]	b (*)	a (*)	a (*)	a (**)	a (*)	c (*)	a (*)	8
4	Ju, 2021 [36]	b (*)	a (*)	c	a (**)	a (*)	c (*)	a (*)	7
5	Hamzehgardeshi, 2020 [30]	b (*)	a (*)	c	a (**)	a (*)	c (*)	a (*)	7
6	Cerentini, 2020 [41]	b (*)	b	a (*)	a (**)	a (*)	c (*)	a (*)	7
7	Rezaie-Chamani, 2020 [32]	b (*)	b (*)	c	a (**)	a (*)	c (*)	a (*)	7
8	Zheng, J, 2020 [19]	a (*)	a (*)	a (*)	a (**)	a (*)	c (*)	a (*)	8
9	Yilmaz, 2020 [40]	b (*)	a (*)	a (*)	a (**)	a (*)	c (*)	a (*)	8
10	Butt, 2019 [43]	b (*)	a (*)	c	a (**)	a (*)	c (*)	a (*)	7
11	El-Kashif, 2018 [39]	a (*)	a (*)	c	a (**)	a (*)	c (*)	a (*)	7
12	Ahmed, 2017 [38]	b (*)	a (*)	c	a (**)	a (*)	c (*)	a (*)	7
13	Lou, 2017 [37]	b (*)	a (*)	a (*)	a (**)	a (*)	c (*)	a (*)	8
14	Alidost, 2017 [33]	b (*)	a (*)	c	a (**)	a (*)	c (*)	a (*)	7
15	Shittu, 2017 [44]	b (*)	a (*)	c	a (**)	b (*)	c (*)	a (*)	7
16	Jafarzadeh, 2016 [8]	b (*)	a (*)	c	a (**)	a (*)	c (*)	a (*)	7
17	Javadifar, 2016 [34]	a (*)	a (*)	c	a (**)	a (*)	c (*)	a (*)	7
18	Zhang, 2015 [45]	a (*)	a (*)	c	a (**)	a (*)	c (*)	a (*)	7
19	Sadat, 2015 [35]	b (*)	a (*)	c	a (**)	a (*)	c (*)	a (*)	7

Selection: (Maximum 5 stars)

1) Representativeness of the sample

a) Truly representative of the average in the target population * (all subjects or random sampling)

b) Somewhat representative of the average in the target population. *(non-random sampling)

c) Selected group of users

d) No description of the sampling strategy

2) Sample size:

a) Justified and satisfactory*

b) Not justified

3) Non-respondents:

a) Comparability between respondents’ and non-respondents characteristics is established, and the response rate is satisfactory*

b) The response rate is unsatisfactory, or the comparability between respondents and non-respondents is unsatisfactory

c) No description of the response rate or the characteristics of the responders and the non-responders

4) Ascertainment of the exposure:

a) Validated measurement tool**

b) Non-validated measurement tool, but the tool is available or described*

c) No description of the measurement tool

Comparability: (Maximum 2 stars)

1) The subjects in different outcome groups are comparable based on the study design or analysis. Confounding factors are controlled

a) The study controls for the most important factor (select one) *

b) The study control for any additional factor*

Outcome: (Maximum 3 stars)

1) Assessment of the outcome:

a) Independent blind assessment**

b) Record linkage**

c) Self-report*

d) No description

2) Statistical test:

a) The statistical test used to analyze the data is clearly described and appropriate, and the measurement of the association is presented, including confidence intervals and the probability level (p-value) *

b) The statistical test is not appropriate, not described, or incomplete

**Table 5:** Methodological quality assessment through NEWCASTLE – OTTAWA scale (for cohort studies)

Row	First author/year	Selection				Comparability	Outcome			Scoring
		Representativeness of the sample	Selection of the non-exposed cohort	Ascertainment of exposure	Demonstration that outcome of interest was not present at the start of the study	The subjects in different outcome groups are comparable based on the study design or analysis. Confounding factors are controlled	Assessment of the outcome	Was follow-up long enough for outcomes to occur	Adequacy of follow-up of cohorts	
1	Loy, 2021 [42]	b (*)	a (*)	a (*)	a (*)	a (*)	b (*)	a (*)	a (*)	Good

## Discussion

This study aimed to systematically review the prevalence and risk factors of FSD and SD among reproductive-aged women. The literature review showed that various studies had evaluated the FSD in multiple cultures, populations, and settings. FSD is a multi-causal and multi-dimensional medical problem that can negatively affect the couple’s fertility status and quality of life and may lead to a decrease in self-esteem and disrupted interpersonal and intrapersonal relationships [47, 48]. The results of this systematic review indicated that 18 of 20 studies reported the prevalence of FSD among reproductive-aged women, so FSD ranged between 20.6% and 95.0%. Based on a systematic review and meta-analysis that assessed the Iranian published studies regarding the prevalence of sexual dysfunction among reproductive-aged women, 52% (95% CI: 39–66%) of women had FSD [22]. The results of another systematic review and meta-analysis demonstrated that the prevalence of sexual dysfunction among reproductive-aged was estimated at 50.75% (95% CI: 41.7–59.7) [10]. Although the mentioned studies had similar primary outcomes to this systematic review, this study assessed all studies regarding FSD among healthy reproductive-aged women until now. Regarding the domains of FSFI, the ranges of all domains were very wide due different settings (clinic-based vs. population-based studies may yield different prevalence rates e.g., clinical settings may yield higher prevalence rates due to comorbidities) and culture (more conservative cultures may underreport because of stigmatization, and more liberal communities may have more rates). Although the measurement tool in most of these studies was FSFI, these differences in reported prevalence can be due to differences in cut-off points, translations, or adaptations, the different sample sizes and sampling methods.

The results of this study showed that the prevalence of SD was assessed in four studies and ranged between 31.8% and 83.4% in reproductive-aged women which highlighting a significant gap in research on this critical aspect of FSD. This wide range suggests substantial variability, which may be attributed to differences in study populations, cultural contexts, and measurement methodologies. The results of a study which investigated the prevalence and associated factors of sexual problems and distress in United States women showed that the prevalence of any sexual problem was 43.1% and also 22.2% of the participants for sexually related personal distress [49].

This research identified a lower prevalence of sexually related personal distress than in the range present in this review. Differences in sample characteristics may be responsible for this difference, and in population-based surveys milder cases may be identified, whereas clinical or high-risk population studies (e.g., women with chronic FSD symptoms) may identify more distress rates. Until recently, there was no systematic review to determine the prevalence of SD among reproductive-aged women. In addition, few epidemiologic studies have assessed SD as a sexual disorder among different populations. Hence, the exact prevalence of this problem and its associated factors have been less studied. The new clinical guidelines in this regard indicated that the presence of distress as a result of FSD is necessary for the diagnosis of this disorder [19, 50]. All four studies that reported the prevalence of SD among reproductive-aged women used FSDS-R with similar standard cut-off points to measure SD [19, 31, 38, 41]. Overall, the higher SD prevalence in some studies may reflect cultural factors, such as restrictive sexual norms or lack of sexual education, which exacerbate distress. In contrast, lower prevalence of SD may be due to greater openness in discussing sexual health or better access to therapeutic interventions.

The results of this study also indicated that various factors lead to FSD and SD among reproductive-aged women. These factors were classified into five main categories for FSD and four main categories for SD. This study’s results also indicated that 17 studies of 20 reported the associated factors of FSD, and only two mentioned the associated factors of SD. As reported in this study, sociodemographic factors such as higher BMI, higher age, higher marriage duration, rural residential environment, primary or secondary educational status, smoking, no specified spouse’s employment status, level of income satisfaction were the most assessed risk factors of FSD among reproductive-aged women. So, reproductive health specialists and sexologists are important to evaluate these factors in women with FSD complaints. The results of a systematic review that assessed the predictors of FSD through gender inequality paradigms showed that factors such as poor physical and mental health, female genitourinary and medical problems, being religious, history of sexual abuse, and relationship dissatisfaction were the main associated factors of the FSD across the world. This study showed that the risk factors of FSD were significantly different based on the study’s location and cultural context [13]. Notably, our focus on reproductive-aged women contrasts with mentioned review which included all age groups and found menopausal status to be a dominant predictor. Another systematic review that assessed FSD among diabetic women showed that depression, anxiety, and duration of diabetes were significantly associated with FSD among premenopausal women. This systematic review and meta-analysis assessed premenopausal women with type 1 diabetes [51] while this current systematic review assessed healthy women. Overall, the variability in risk factors by geography highlights the role of cultural context. Reproductive health specialists should adopt tailored screening tools for instance, prioritizing socioeconomic stressors in resource-limited areas and mental health in urbanized populations.

The results of this study also indicated that some factors such as sociodemographic factors included satisfaction with marriage, spouse’s premature ejaculation disorder, pain during sexual intercourse and sexual inactivity lead to SD among reproductive-aged women which reported by only two mentioned included studies women [19, 31]. The results of a study which investigated the associated factors of SD among women indicated that sociodemographic factors such as poor self-assessed health, lower educational level, psychological factors included depression, anxiety, and medical factors such as thyroid conditions, and urinary incontinence are correlated with SD among women [49]. Unlike mentioned study, which highlighted thyroid disorders and urinary incontinence, our included studies did not extensively examine these medical contributors. This discrepancy may stem from differences in study populations or measurement tools, suggesting that future research should standardize assessments of medical comorbidities in SD. According to results of an Iranian study which assessed the predictor factors of SD in women demonstrated that FSD is the most important predictor of SD. In addition, longer marriage duration, dissatisfaction relationship with the spouse and higher rate of depression, anxiety and stress were associated with SD among women [52]. The results of mentioned studies were consistent with this current study. The mentioned Iranian study placed stronger emphasis on marital duration and dissatisfaction, whereas studies from Western populations [49], prioritized individual mental health. This divergence underscores the need for culturally adapted interventions. Overall, clinicians should evaluate both individual (mental health, medical conditions) and relational (marital quality, partner sexual health) factors when diagnosing SD in reproductive-aged women.

### Strengths and limitations

To our knowledge, this systematic review is the first study that assessed FSD and SD among healthy reproductive-aged women and included all of the studies among healthy reproductive-aged women. As in some of the included studies, the odds ratio of the related factors is not reported, so we only report their p-value to show the significant effect in the tables. The language restriction to Persian and English languages is another limitation of this study.

## Conclusions

The results of this study showed that reproductive-aged women are at high risk of FSD and SD due to sociodemographic, reproduction, interpersonal, psychological, and medical factors. Although multiple factors were associated with the prevalence of FSD and SD, the participants’ sociodemographic characteristics were the main factors associated with them. As the literature regarding SD among the reproductive-aged population was limited, conducting high-quality cross-sectional studies with representative samples and using validated questionnaires are required to provide more precise data regarding its prevalence and associated factors worldwide. In addition, conducting epidemiological studies in this regard among women with chronic physical and psychological diseases are proposed.

## Supplementary Information

Supplementary Material 1.

## Data Availability

The datasets used and/or analyzed during the current study available from the corresponding author on reasonable request.

## Abbreviations

FSD: Female sexual dysfunction
SD: Sexual distress
SDD: Sexual desire disorder
STDs: Sexually transmitted diseases
BMI: Body mass index
NVD: Normal vaginal delivery
OR: Odds ratio
