# Supplementary material for: Prevalence and correlates of female sexual dysfunction and sexual distress in reproductive-aged women: a systematic review and meta-analysis
Source: BMC Womens Health. 2025 Sep 29;25:451. doi: 10.1186/s12905-025-03960-4 (PMC12481727; doi:10.1186/s12905-025-03960-4)
Supplement: Supplementary file 1 — Supplementary Material 1. [file 12905_2025_3960_MOESM1_ESM.pdf]

SPRINGER NATURE

Change of authorship request form - Journals (pre-acceptance)

Important information. Please read.

- This form should be used by authors to request any change in authorship (adding/deleting authors) including changes in corresponding authors. This form should not be used for name changes. Please fully complete all sections. Use black ink and block capitals and provide each author's full name with the given name first followed by the family name.

- By signing this declaration, all authors guarantee that the order of the authors are in accordance with their scientific contribution, if applicable as different conventions apply per discipline, and that only authors have been added who made a meaningful contribution to the work.

- Please note, in author collaborations where there is formal agreement for representing the collaboration, it is sufficient for the representative or legal guarantor (usually the corresponding author) to complete and sign the Authorship Change Form on behalf of all authors, **next to the added/removed author(s)**. **(Complete Section 3, followed by Section 6.)**
  In author collaborations where there is no formal agreement for representing the collaboration and **there are more than 10 authors**, one may sign for all, provided the signer appends correspondence that attests that each of the authors have agreed to the change **and the added/removed authors sign the form**. **(Complete Section 3, followed by Section 6.)**

- Please note, we cannot investigate or mediate any authorship disputes. If you are unable to obtain agreement from all authors (including those who you wish to be removed) you must refer the matter to your institution(s) for investigation. Please inform us if you need to do this.

- If you are not able to return a fully completed form within **30 days** of the date that it was sent to the author requesting the change, we may have to withdraw your manuscript. We cannot publish manuscripts where authorship has not been agreed by all authors (including those who have been removed).

- Incomplete forms will be rejected.

- Please return/upload this form, fully completed, to the Journals Editorial Office. The Journal and/or Publisher will consider the information you have provided to decide whether to approve the proposed change in authorship. We may decide to contact your institution for more information or undertake a further investigation, if appropriate, before making a final decision.

SPRINGER NATURE

Change of authorship request form - Journals (pre-acceptance)

Section 1: Please provide the current title of manuscript

Manuscript ID no.: d8f1eede-ac3a-4a56-8875-d4a82f0a6086

Title: The prevalence and related factors of sexual dysfunction and sexual distress among healthy reproductive-aged women: A systematic review and meta-analysis of observational studies

Section 2: Please provide the previous authorship, in the order shown on the manuscript before the changes were introduced. Please indicate the corresponding author by adding (CA) behind the name.

	First name(s)	Family name	ORCID or SCOPUS id, if available
1st author	Fatemeh	Heshmatnia	
2nd author	Marzieh	Azizi	https://orcid.org/0000-0002-0835-3201
3rd author	Hamed	Milani	
4th author	Mohsen	Kheiri	
5th author	Hadis	Tolomehr	
6th author	Zohreh	Shahhosseini (corresponding author)	https://orcid.org/0000-0003-1288-2277
7th author			
8th author			
9th author			
10th author			

Please use an additional sheet if there are more than 10 authors.

SPRINGER NATURE Springer Nature is one of the world's leading global research, educational and professional publishers, created in May 2015 through the combination of Nature Publishing Group, Palgrave Macmillan, Macmillan Education and Springer Science+Business Media.

SPRINGER NATURE

Change of authorship request form - Journals (pre-acceptance)

Section 3: Please provide a justification for change. Please use this section to explain your reasons for changing the authorship of your manuscript, e.g. what necessitated the change in authorship? Please refer to the (journal) policy pages for more information about authorship. Please explain why omitted authors were not originally included and/or why authors were removed on the submitted manuscript.

After the valuable comments of the reviewers regarding the meta-analysis of the prevalence section of this study, we used the consultation of a statistical expert and **"Dr Roya Nikbakht**" performed this section. In addition, she checked all section of this study regarding its accuracy and edit, therefore the authors of this study decided to add her as a author.
In addition, **Dr Marzieh Azizi** is considered as **a secondary corresponding author** of this manuscript. All authors accepted this decision.

Section 4: Proposed new authorship. Please provide your new authorship list in the order you would like it to appear on the manuscript. Please indicate the corresponding author by adding (CA) behind the name. If the Corresponding Author has changed, please indicate the reason under section 3.

	First name(s)	Family name (this name will appear in full on the final publication and will be searchable in various abstract and indexing databases)	Affiliated institute	E-mail address
1st author	Fatemeh	Heshmatnia	Department of Nursing and Midwifery, Semnan Branch, Islamic Azad University, Semnan, Iran	fatemeh.heshmatnia.1994@gmail.com
2nd author	Marzieh	Azizi **(secondary corresponding author)**	Department of Midwifery, Sexual and Reproductive Health Research Center, Mazandaran University of Medical Sciences, Sari, Iran	marziehazizi70@gmail.com
3rd author	Hamed	Milani	School of Medicine, Mazandaran University of Medical Sciences, Sari, Iran	dr.hamedmilani@yahoo.com
4th author	Roya	Nikbakht	Department of Biostatics and Epidemiology, Faculty of Health, Mazandaran University of Medical Sciences, Sari, Iran	roya.nikbakht2011@gmail.com
5th author	Mohsen	Kheiri	Student Research Committee, School of Pharmacology, Mazandaran University of Medical Sciences, Sari, Iran	kheirimohsen80@gmail.com
6th author	Hadis	Tolomehr	Student Research Committee, School of Paramedical Sciences, Mazandaran University of Medical Sciences, Sari, Iran	hadistolomehr@gmail.com
7th author	Zohreh	Shahhosseini **(Primary corresponding author)**	Department of Midwifery, Sexual and Reproductive Health Research Center, Mazandaran University of Medical Sciences, Sari, Iran	zshahhosseini@yahoo.com
8th author				

Please use an additional sheet if there are more than 10 authors.

SPRINGER NATURE Springer Nature is one of the world's leading global research, educational and professional publishers, created in May 2015 through the combination of Nature Publishing Group, Palgrave Macmillan, Macmillan Education and Springer Science+Business Media.

Change of authorship request form - Journals (pre-acceptance)

Section 5: Author contribution, Acknowledgement and Disclosures. Please use this section to provide a new disclosure statement and, if appropriate, acknowledge any contributors who have been removed as authors and ensure you state what contribution any new authors made (if applicable per the journal or book (series) policy). Please ensure these are updated in your manuscript - after approval of the change(s) - as our production department will not transfer the information in this form to your manuscript.

New acknowledgements:
Not applicable

New Disclosures (financial and non-financial interests, funding):
Not applicable

New Author Contributions statement (if applicable per the journal policy):
R.N.: Conducted the meta-analysis of this study

SPRINGER NATURE Springer Nature is one of the world's leading global research, educational and professional publishers, created in May 2015 through the combination of Nature Publishing Group, Palgrave Macmillan, Macmillan Education and Springer Science+Business Media.

Change of authorship request form - Journals (pre-acceptance)

State 'Not applicable' if there are no new authors.

Section 6: Declaration of agreement. *All* authors, unchanged, new and removed *must* sign this declaration.
(NB: Please print the form, (docu)-sign and return/upload a scanned copy. Please note that signatures that have been inserted as an image file are acceptable as long as it is handwritten. Typed names in the signature box are unacceptable.) *** Please delete as appropriate. Delete all of the bold if you were on the original authorship list and are remaining as an author.**

	First name	Family name		Signature	Date
1st author	Fatemeh	Heshmatnia	I agree to the proposed new authorship shown in section 4 **/and the addition/removal*of my name to the authorship list** /and the proposed change in corresponding author		27 Jan 2025
2nd author	Marzieh	Azizi	I agree to the proposed new authorship shown in section 4 **/and the addition/removal*of my name to the authorship list** /and the proposed change in corresponding author		27 Jan 2025
3rd author	Hamed	Milani	I agree to the proposed new authorship shown in section 4 **/and the addition/removal*of my name to the authorship list** /and the proposed change in corresponding author		27 Jan 2025
4th authors	Roya	Nikbakht	I agree to the proposed new authorship shown in section 4 **/and the addition/removal*of my name to the authorship list** /and the proposed change in corresponding author		27 Jan 2025
5th author	Mohsen	Kheiri	I agree to the proposed new authorship shown in section 4 **/and the addition/removal*of my name to the authorship list** /and the proposed change in corresponding author		27 Jan 2025
6th author	Hadis	Tolomehr	I agree to the proposed new authorship shown in section 4 **/and the addition/removal*of my name to the authorship list** /and the proposed change in corresponding author		27 Jan 2025

SPRINGER NATURE Springer Nature is one of the world's leading global research, educational and professional publishers, created in May 2015 through the combination of Nature Publishing Group, Palgrave Macmillan, Macmillan Education and Springer Science+Business Media.

SPRINGER NATURE

Change of authorship request form - Journals (pre-acceptance)

7th author	Zohreh	Shahhosseini	I agree to the proposed new authorship shown in section 4 /**and the addition/removal*of my name to the authorship list** /and the proposed change in corresponding author	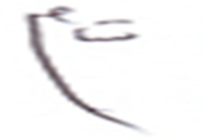	27 Jan 2025

SPRINGER NATURE Springer Nature is one of the world's leading global research, educational and professional publishers, created in May 2015 through the combination of Nature Publishing Group, Palgrave Macmillan, Macmillan Education and Springer Science+Business Media.

SPRINGER NATURE

Change of authorship request form - Journals (pre-acceptance)

	First name	Family name		Signature	Date
8th author			I agree to the proposed new authorship shown in section 4 /**and the addition/removal*of my name to the authorship list** /and the proposed change in corresponding author		
9th author			I agree to the proposed new authorship shown in section 4 /**and the addition/removal*of my name to the authorship list** /and the proposed change in corresponding author		
10th author			I agree to the proposed new authorship shown in section 4 /**and the addition/removal*of my name to the authorship list** /and the proposed change in corresponding author		

Please use an additional sheet if there are more than 10 authors.

In case of author collaborations with formal agreement:

	Name of consortium/consortia	First name	Family name		Signature	Date
Representative/legal guarantor				I agree to the proposed new authorship shown in section 4 /**and the addition/removal*of my name to the authorship list** /and the proposed change in corresponding author		

Both added/removed authors should complete the information in the first table under Section 6.

---- End of form ----

SPRINGER NATURE Springer Nature is one of the world's leading global research, educational and professional publishers, created in May 2015 through the combination of Nature Publishing Group, Palgrave Macmillan, Macmillan Education and Springer Science+Business Media.
